# Client risk segmentation to identify and respond to the differentiating characteristics of clients with advanced HIV disease in Kyrgyzstan: A retrospective analysis of routine program data

**DOI:** 10.1097/MD.0000000000043251

**Published:** 2025-07-04

**Authors:** Aisuluu Kubatova, Talgat Mambetov, Christa Fischer Walker, Amy Gottlieb, Olga Samoilova, Daniiar Saliev, Michael M. Cassell

**Affiliations:** a Strategic Information, FHI360, EpiC, Bishkek, Kyrgyzstan; b Strategic Information, FHI360, Washington, DC.

**Keywords:** advanced HIV disease, ART, HIV, Kyrgyzstan

## Abstract

The World Health Organization recommends a comprehensive package of interventions to screen for, prevent, and treat advanced human immunodeficiency virus (HIV) disease (AHD). However, implementation remains suboptimal, particularly among key populations. We conducted a retrospective analysis of routine clinical data from HIV treatment sites supported by the PEPFAR-funded USAID Meeting Targets and Maintaining Epidemic Control (EpiC) Kyrgyzstan project. The analysis included baseline demographic and clinical characteristics of newly diagnosed adult persons living with HIV who initiated antiretroviral therapy between September 1, 2020 and December 31, 2021, at EpiC-supported sites. AHD was defined as a CD4 cell count <200 cells/mm or meeting the World Health Organization criteria for stage 3 or 4 disease. We used bivariate and multivariable logistic regression models to examine associations between client characteristics and AHD. Among 240 clients, 79 (32.9%) presented with AHD. In bivariate analysis, AHD was significantly associated with age ≥ 30 years (OR = 4.15; CI = 1.93–8.92); being widowed or divorced (OR = 3.21; CI = 1.78–5.78); or being a person who injects drugs, a sexual partner of persons living with HIV or client of female sex workers (OR = 4.44; CI = 2.13–9.25). In multivariable analysis, all except age remained significant predictors. Routine program data can help identify high-risk groups for AHD. Segmentation based on client characteristics may inform strategies to promote earlier HIV testing and tailored interventions for prevention and care.

## 1. Introduction

Advanced HIV disease (AHD), defined as a CD4 cell count <200 cells/mm or World Health Organization (WHO) stage 3 or 4 disease, is associated with morbidity and mortality among people living with human immunodeficiency virus (PLHIV), even among those receiving antiretroviral treatment (ART).^[1]^ Individuals with AHD are at high risk for opportunistic infections, such as tuberculosis and cryptococcal meningitis.^[2]^ Management of AHD requires differentiated diagnostics and clinical care beyond routine HIV testing and treatment.^[3]^

Although WHO recommends guidelines for AHD screening, prevention, and treatment, implementation remains limited among key populations (KP).^[4]^ We conducted a retrospective analysis of routine program data collected in EpiC-supported HIV treatment sites in Kyrgyzstan to identify which clients are most likely to present with AHD at diagnosis and to inform public health strategies.

## 2. Methods

### 2.1. Study design and participants

This is a retrospective analysis of de-identified, routine monitoring data collected from HIV care and treatment sites supported by the PEPFAR-funded USAID EpiC project in Kyrgyzstan. These community-based facilities offer HIV prevention, testing, and ART services. We included adults (>18 years of age) newly diagnosed with HIV between September 1, 2020 and December 31, 2021, who initiated ART at EpiC-supported treatment centers. Detailed demographic data was not collected for testing clients, so those who did not initiate ART were excluded. We extracted de-identified individual-level data from the EpiC DHIS2-tracker and the Republican AIDS Center electronic databases. These data were collected as part of routine program monitoring activities. The analysis was conducted for quality improvement and was not intended to test a specific scientific hypothesis.

### 2.2. Statistical analysis

We calculated the proportion of individuals presenting with AHD at diagnosis using the WHO definition of a documented CD4 cell count < 200 cells/mm or clinical stage 3 or 4 based on clinical assessment.^[5]^ Associations between socio-demographic characteristics of the client and presence of AHD at time of diagnosis were determined using a bivariate logistic regression model. We categorized divorced and widowed together because it was thought that these clients were more likely to have had a partner who had had HIV but had not had an immediate HIV test/diagnosis. We categorized age as younger/older than 30 years of age to dichotomize younger versus older adults. We created a dichotomous population variable after exploratory analysis of all included populations and the proportion presenting with AHD. Variables considered statistically significant (*P* < .10) were then included in a multivariable logistic regression model. All analyses were conducted using Wizard Pro.^[6]^

## 3. Results

**Figure 1.**
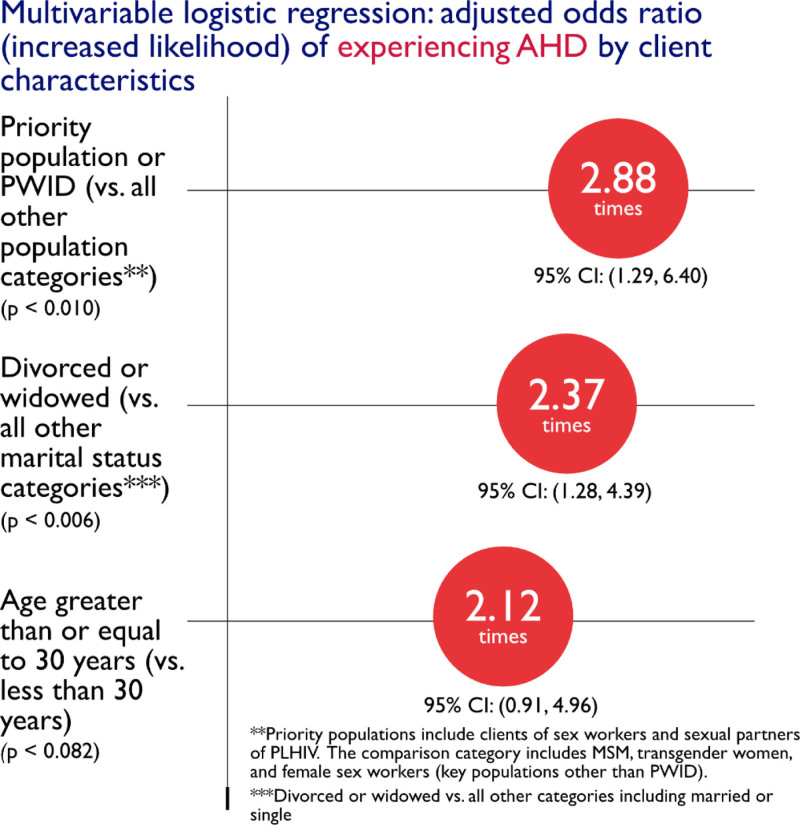
Multivariable analysis of client characteristics associated with AHD at time of HIV diagnosis. AHD = advanced HIV disease.

EpiC identified 252 newly diagnosed PLHIV from September 1, 2020, to December 31, 2021. Of these, 240 started ART and were included in the analysis. Twelve individuals who did not start ART were excluded. Seventy-nine of the 240 clients (32.9%) met the definition for AHD at time of diagnosis.^[5]^ Bivariate analyses (Table 1) showed AHD was significantly more likely among clients ≥ 30 years old (OR: 4.15; 95% CI: 1.92–8.92), widowed or divorced (OR: 3.21; 95% CI: 1.78–5.78) and PWID, sexual partners of PLHIV, or clients of sex workers (OR: 4.44; 95% CI: 2.13–9.25). In the multivariable model, only population group and marital status remained statistically significant (Fig. 1).

**Table 1:** Bivariate analysis of client characteristics and the odds of presenting with AHD at time of diagnosis.

Variables		At time of HIV diagnosis		OR (95% CI)
		Presenting without AHD (n = 161) n (%)	Presenting with AHD (n = 79) n (%)	
Gender	F	55 (65.5)	29 (34.5)	0.89 (0.51–1.57)
	M	106 (68.0)	50 (32.1)	
Age	≥30 yr old	105 (60.0)	70 (40.0)	**4.15 (1.93–9.92**)
	<30 yr old	56 (86.2)	9 (13.8)	
Education	Secondary education or below	121 (63.7)	69 (35.8)	1.98 (0.95–4.11)
	Beyond secondary level	39 (77.8)	11 (22.2)	
Marital status	Widowed/divorced	32 (47.8)	35 (52.2)	**3.21 (1.78–5.78**)
	Married/single	129 (74.6)	44 (25.4)	
Citizenship	Kyrgyzstan	148 (67.0)	73 (33.0)	0.94 (0.34–2.56)
	Other	13 (68.4)	6 (31.6)	
Population group	Priority population or PWID	98 (58.7)	69 (41.3)	**4.44 (2.13–9.25**)
	Other population groups	63 (86.3)	10 (13.7)	
Employment status	Yes	44 (73.3)	16 (26.7)	0.68 (0.35–1.29)
	No	117 (65.0)	63 (35.0)	
Labor migration past\present	Yes	77 (68.8)	35 (31.3)	0.85 (0.49–1.49)
	No	73 (65.2)	39 (34.8)	
Testing modality	Index testing	47 (58.8)	33 (41.3)	1.74 (0.99–3.05)
	Other	114 (71.3)	46 (28.8)	

Bivariate analysis of client characteristics and the odds of presenting with advanced HIV disease at time of diagnosis. Bold values indicate statistically significant results (*P* < .05). AHD = advanced HIV disease, CI = confidence interval, OR = odds ratio, PWID = people who inject drugs.

## 4. Discussion

Using routinely collected client data from a cohort of newly diagnosed and initiated PLHIV in Kyrgyzstan we identified characteristics of clients presenting with AHD upon HIV diagnosis. Among clients newly identified HIV positive, we found that those who were sexual partners of sex workers or PLHIV, PWID, and those divorced or widowed were more likely to present with AHD compared to clients without these characteristics. Previous studies conducted in Cameroon, Botswana, and China also found similar risk groups, particularly identifying as a KP, notably PWID; older age; lower educational attainment; and being divorced or widowed as significant risk factors associated with AHD.^[3,7–10]^ Several studies highlight the importance of targeted HIV programming for KP, such as PWID, which aligns with the findings of our analysis.^[11]^

Client risk segmentation, as presented in this analysis, has neither the intent nor the capacity to establish causal relationships between client characteristics and health outcomes. Rather, it aims to provide actionable insights into the characteristics of clients at heightened risk. Preventing morbidity and premature mortality from HIV necessitates the early identification of HIV infections prior to AHD onset and the timely treatment. Our model suggests that high-risk population groups can be identified through the analysis of historical data, which could then be utilized to enhance the efficiency and effectiveness of targeted HIV testing programs.^[12]^

Using historical program data, the model can be used to generate predictions of the likelihood of clients presenting with AHD. For example, the model predicts that clients younger than 30 years, who are neither PWID nor members of a priority populations, and not divorced or widowed, have an 8.5% chance of presenting AHD. Conversely, clients who are 30 years or older, PWID or member of a priority population, and divorced or widowed, have a predicted 57.3% likelihood of presenting with AHD.

The results of this analysis informed stakeholders in Kyrgyzstan and supported programmatic changes geared toward identifying older clients, PWID, and priority populations. Enhanced awareness and targeted testing approaches for these high-risk groups led to an observed reduction in the proportion of clients presenting with AHD at ART initiation, from 32% in 2022 to 27% in 2023.

However, this analysis had several limitations. Firstly, the use of routine programmatic data restricts the application of findings strictly to public health program improvements rather than to individual clinical decision making. Additionally, the model relies on a limited number of historical observations and thus requires validation and continuous updating as the program expands and more comprehensive data becomes available. Given these limitations, further research involving larger patient cohorts and more diverse datasets is essential. Additional clinical studies are necessary to confirm the observed associations and refine the predictive accuracy of the model. Expanding research efforts to include prospective studies would improve the understanding of causal relationships and help validate findings in varied population settings. Ultimately, sustained investment in robust data collection and analysis infrastructure, coupled with larger-scale clinical studies, will be critical to effectively reducing the burden of AHD in vulnerable and high-risk communities.

## 5. Conclusion

Our analysis demonstrates that routinely collected programmatic data can identify groups at elevated risk for presenting with AHD at diagnosis. Targeting older clients, PWID, divorced or widowed individuals, and partners of KP can significantly enhance HIV testing efficiency and reduce late presentation. However, validation through further studies with larger cohorts and diverse datasets remains essential. Continued investment in comprehensive data collection and analysis is critical to reducing the burden of AHD and improving HIV outcomes in vulnerable populations.

## Acknowledgments

We would like to thank the staff of EpiC Kyrgyzstan and the Republican Center for Blood Born Viral Hepatitis and HIV Control of Kyrgyz Republic for project support. EpiC is funded by the United States President’s Emergency Plan for AIDS Relief (PEPFAR) through the Family Health International (FHI 360). The findings and conclusions in this report are those of the author(s) and do not necessarily represent the official position of USAID or FHI 360. Preliminary results were presented at the Asia Pacific AIDS and Co-infections Conference (APACC), Singapore, June 8 to 10, 2023.

## Author contributions

**Conceptualization:** Talgat Mambetov, Amy Gottlieb, Michael M. Cassell.

**Data curation:** Talgat Mambetov, Michael M. Cassell.

**Formal analysis:** Aisuluu Kubatova, Michael M. Cassell.

**Funding acquisition:** Michael M. Cassell.

**Investigation:** Aisuluu Kubatova, Christa Fischer Walker, Michael M. Cassell.

**Methodology:** Michael M. Cassell.

**Project administration:** Amy Gottlieb, Olga Samoilova, Daniiar Saliev, Michael M. Cassell.

**Supervision:** Christa Fischer Walker, Amy Gottlieb, Olga Samoilova, Michael M. Cassell.

**Validation:** Aisuluu Kubatova.

**Visualization:** Aisuluu Kubatova, Michael M. Cassell.

**Writing – original draft:** Aisuluu Kubatova, Christa Fischer Walker.

**Writing – review & editing:** Aisuluu Kubatova, Christa Fischer Walker, Michael M. Cassell.
